# Predictive factors and outcomes of severe community acquired pneumonia in patients with respiratory failure

**DOI:** 10.12669/pjms.38.4.5312

**Published:** 2022

**Authors:** Arslan Rahat Ullah, Aysha Masood, Sumayya Amin, Iftikhar Ali

**Affiliations:** 1 Dr. Arslan Rahat Ullah, FCPS. Department of Medicine & Allied, Northwest General Hospital & Research Centre, Peshawar, Pakistan; 2 Dr. Aysha Masood, MBBS. Department of Thoracic medicine, Royal Bournemouth Hospital, Castle Ln E, Bournemouth BH7 7DW, United Kingdom; 3 Dr. Sumayya Amin, MBBS. Department of Medicine & Allied, Northwest General Hospital & Research Centre, Peshawar, Pakistan; 4 Iftikhar Ali, MPH. Pharmacy Unit, Paraplegic Centre, Hayatabad, Peshawar, Pakistan

**Keywords:** Severe community-acquired pneumonia, Predictive factors, Mortality, Respiratory failure

## Abstract

**Objectives:**

To explore the risk factors, pathogens and outcomes of severe community-acquired pneumonia (SCAP) in patients with respiratory failure.

**Methods:**

A prospective observational study was conducted at Northwest General Hospital & Research Centre, Peshawar, Pakistan from February 2016 to October 2018. All patients with Community-acquired pneumonia (CAP) who fulfilled the inclusion criteria were recorded consecutively. Diagnosis of SCAP was made following the criteria established by the IDSA/ATS in the consensus guidelines on the management of CAP in adults published in 2007. In-hospital mortality was the main outcome.

**Results:**

The final analysis comprised a total of 100 patients with SCAP. The mean age was 60.0±18.01 years, and 54.0% were female patients. Afghani patients represented 22.0% of the total patients. The most common comorbidity associated with SCAP was hypertension (42.0%). The most commonly isolated etiological agents were Acinetobacter baumannii, followed by extended-spectrum beta-lactamase (ESBL)-producing Escherichia coli. In-hospital mortality was 45%. On multivariate analysis, factors associated with in-hospital mortality were age (OR 1.054; 95%Cl 1.01-1.10; p=0.021), presence of two or more complications (OR 4.51; 95%Cl 1.18-17.28; p=0.028), septic shock (OR 6.44; 95%Cl 1.55-26.803; p=0.010), length of mechanical ventilation (OR 1.17; 95%Cl 1.01-1.40; p=0.043), and paO_2_ (OR 4.51; 95%Cl 1.18-17.28; p=0.004).

**Conclusion:**

A high mortality rate was observed in our study. Age, presence of two or more complications, septic shock, length of mechanical ventilation, and low paO_2_ were identified to be independent predictors of mortality for patients with SCAP.

## INTRODUCTION

Community-acquired pneumonia (CAP) is a frequent and fatal infection. Despite breakthroughs in antimicrobial therapy and supportive approaches, CAP lingers on to be a prime concern and contributes to substantial morbidity and mortality globally. Severe community-acquired pneumonia (SCAP) typically necessitates hospitalization and intensive care. SCAP or CAP that requires intensive care unit (ICU) admission portends a noticeably serious prognosis.^1^-^3^ The severity of illness, certain patient characteristics and co-comorbidities in these patients are typically related to poor prognosis.^4^ SCAP is defined as the presence of acute respiratory failure (ARF) needing supportive therapy and/or septic shock with multi-organ dysfunction.^5^,^6^ It has a high mortality ranging from 18% in non-ventilated patients to 33% in mechanically ventilated patients.^7^ An initial assessment of all patients with CAP to guide management and early decision-making regarding the need for hospital admission or intensive care is imperative in reducing the frequency of deaths.

The factors affecting mortality in these patients in the ICU are composite. The main risk factors are advanced age, the presence of malignancy, multilobar consolidation, ARF, high lactate level, inadequate antimicrobial therapy, invasive mechanical ventilation (IMV), multi-organ system dysfunction, altered mental status, a high acute physiology and chronic health evaluation (APACHE) II score and high NT-pro BNP (N-terminal pro b-type natriuretic peptide) levels.^7^-^10^

Patients with respiratory failure are associated with high in-hospital mortality.^11^,^12^ Such patients may require IMV, which is linked to a wide variety of complications and a high mortality rate.^7^,^13^,^14^ Regardless of whether or not IMV is used, the need for it can be a sign of more serious acute disease. In this part of the world, however, no prospective studies have been performed on the predictive factors and outcomes have not been well investigated among patients with SCAP who are in respiratory failure. In view of the paucity of data on this subject in Pakistan, we aimed to explore the risk factors, pathogens and outcomes associated with SCAP.

## METHODS

A prospective observational study was conducted at Northwest General Hospital & Research Centre, which is a tertiary care hospital based in Peshawar’s historic City Centre. Patients with CAP admitted in the medical ICU were prospectively reported and tracked before discharge or death. The admitting physician/intensivist established the diagnosis of CAP in accordance with the “Infectious Diseases Society of America/American Thoracic Society Consensus Guidelines on the Management of Community-Acquired Pneumonia in Adults published in 2007”.^1^ A total of 278 patients with pneumonia were examined and 146 patients were considered for inclusion. Of these, 100 patients were finally enrolled in our study. All patients 16 years of age or older with SCAP were considered. Patients excluded from the study were those who had less than 24 hours ICU stay, about whom there was an absence of essential data, whose age was less than 16 years, and patients with a hospital-acquired pneumonia, suspected or confirmed tuberculosis and aspiration pneumonia. A structured format was used for data collection. The data collected included age, gender, nationality, clinical characteristics comorbidities, length of hospital and ICU stay, chest radiographic features, mental status, and laboratory findings, APACHE II score, presence of septic shock, the need for mechanical ventilation (MV), duration of MV, micro-organisms, presence of renal impairment, the need for dialysis and outcome. The microbiological data collected in our study comprised of sputum, bronchoalveolar lavage, blood and body fluid samples sent for culture and sensitivity. Additionally, sputum samples were also sent for acid-fast bacilli (AFB) smear and mycobacterial culture and sensitivity. The hospital’s ethical review committee approved the study. (Ref. No.: NwGH/DMER/EC/2516).

Data analysis was carried out using SPSS version 21. The mean (SD) or median with interquartile range (IQR) was calculated for numerical data where appropriate. Frequencies and percentages were used to express the values for the categorical/ordinal data. The effect of each variable on the outcome was determined using univariate analysis. Variables with a p-value <0.05 were maintained in the final model using a forward stepwise selection procedure. Odds ratios (ORs) and their 95% confidence intervals (CIs) were calculated using logistic regression to investigate multivariate associations. An alpha of 0.05 and a 95% Cl was used to determine statistical significance.

## RESULTS

The mean age of the patients was 60.0±18.01 years. There were 54 (54.0%) female patients and Afghans represented 22 (22.0%) of the total patients. The presence of comorbidities was noted in 90 (90.0%) patients. Hypertension and chronic obstructive airways disease (COPD) were the most common comorbidities associated with SCAP and present in 42 (42%) and 37 (37%) of the patients respectively. Forty-nine percent of patients developed more than one in-hospital complication, with renal impairment being identified in 53% patients, followed by an altered mental status (AMS) in 49% patients. Compared to survivors (56.42±19.22), non-survivors were older (64.38±15.52) and the difference was statistically significant (p=0.027). Similarly, non-survivors ended up having more than one complication (p=0.009). Among the complications, renal impairment (p=0.016), AMS (p<0.001), and thrombocytopenia (p=0.019) were specifically more prevalent (Table-I).

**Table-I:** Patients baseline characteristics and cross-tabulation between survivors and non-survivors.

Characteristics	Total (N=100)	Survivors (N=55)	Non-survivors (N=45)	p-value
Age (years), mean± SD	60.0±18.01	56.42±19.22	64.38±15.52	0.027
***Gender [n (%)]***				
Male	46(46.0%)	27(49.1%)	19(42.2%)	0.548
Female	54(54.0%)	28(50.9%)	26(57.8%)	
***Nationality [n (%)]***				
Afghani	22(22.0%)	14(25.5%)	08(17.8%)	0.468
Pakistani	78(78.0%)	41(74.5%)	37(82.2%)	
***Number of co-morbidities [n (%)]***				
None	10(10.0%)	06(10.9%)	04(8.9%)	0.789
One	27(27.0%)	16(29.1%)	11(24.4%)	
≥2	63(63.0%)	33(60.0%)	30(66.7%)
***Co-morbid conditions /Co-existing illness [n (%)]***				
Diabetes mellitus	36(36.0%)	18(32.7%)	18(40.0%)	0.531
Hypertension	42(42.0%)	23(41.8%)	19(42.2%)	1.000
Chronic obstructive airway diseases	37(37.0%)	20(36.4%)	17(37.8%)	1.000
History of malignancy	02(02.0%)	01(01.8%)	10(02.2%)	1.000
Cardiovascular diseases	36(36.0%)	17(30.9%)	19(42.2%)	0.297
Liver diseases	02(02.0%)	02(03.6%)	0(0)	0.500
***Number of complications [n (%)]***				
One	51(51.0%)	35 (63.6%)	16(35.6%)	0.009
>1	49(49.0%)	20(36.4%)	29(64.4%)	
***Complications [n (%)]***				
Pleural effusion	12(12.0%)	06(10.9%)	06(13.3%)	0.764
Renal impairment	53(53.0%)	23(41.8%)	30(66.7%)	0.016
Pregnancy	04(04.0%)	01(01.8%)	03(06.7%)	0.324
Altered mental status	49(49.0%)	16 (29.1%)	33(73.3%)	<0.001
Thrombocytopenia	32(32.0%)	12 (21.8%)	20(44.4%)	0.019

The laboratory parameters, microbiological data and radiological findings of the patients are given in Table-II. Chest x-ray findings showed involvement of two or more quadrants in a majority of patients. No significant median differences in C-reactive protein (CRP), total leucocytes, lactic acid and paCO2 were observed between survivors and non-survivors. On the other hand, paO2 was lower in non-survivors as compared to survivors (p=0.038). The most commonly isolated etiological agents were A. baumannii followed by ESBL-producing E.coli.

**Table-II:** Laboratory parameters, microbiological data and radiological findings of patients.

Characteristics	Total (N=100)	Survivors (N=55)	Non-survivors (N=45)	p-value
***Chest x-ray [n (%)]***				
1 quadrant	18(18.0%)	11(20.0%)	07(15.6%)	0.494
2 quadrant	54(54.0%)	31(56.4%)	23(51.1%)	
3 quadrant	20(20.0%)	08(14.5%)	12(26.7%)	
4 quadrant	08(08.0%)	05(09.1%)	03(06.7%)	
C-reactive protein (n=86) median (IQR)	10.23(21.0)	6.78(19.0)	12.50(20.0)	0.416
Total leucocytes median (IQR)	15150.0(10050)	15000(7835.0)	16820.0(13825)	0.235
Lactic acid (n=60) median (IQR)	25.50(20.0)	22.65(20.0)	27.50(16)	0.075
Arterial pH mean±SD	7.33±0.12	7.35±0.11	7.32±0.13	0.149
paCO_2_ median (IQR)	40.50(22.0)	38.50(21.0)	45.50(24.0)	0.782
paO_2_ mean± SD	75.84±32.50	81.16±30.31	68.51±29.41	0.038
***Microbiological data [n (%)]***				
Blood culture	18(18.8%)	8(15.09%)	10(23.26%)	0.431
Sputum culture	22(50.0%)	14(50.0%)	08(50.0%)	0.623
Brancholalveolar lavage culture	03(50.0%)	03(75.0%)	0	0.400
Body fluids culture	02(40.0%)	0	02(100%)	1.000

Septic shock was recorded in 54% of patients and this was observed more in non-survivors (n=37, 82.2%, p<0.001).Eighty-five patients (85.0%) needed mechanical ventilation (MV), and the mean duration of MV was 4.34±3.95 days. The mean duration of ICU stay was 5.88±3.72 days and overall in-hospital mortality was 45.0%.Renal replacement therapy was required by 18 (18.0%) patients. Non-survivors had statistically significant differences in mean duration of MV (p=0.001), APACHE II score (p<0.001), and the duration of hospital stay (p=0.005), as shown in Table-III.

**Table-III:** Life support requirement and severity of illness.

Characteristics	Total (100)	Survivors (N=55)	Non-survivors (N=45)	p-value
***Shock [n (%)]***				
Yes	54(54.0%)	17(30.9%)	37(82.2%)	<0.001
No	46(46.0%)	38(69.1%)	08(17.8%)	
***Tracheostomy [n (%)]***				
Yes	3(8.3%)	1 (1.8%)	2 (4.4%)	0.587
No	97(91.7%)	54 (98.2%)	43 (95.6%)	
Mechanical ventilation length (days)means± SD	4.34±3.95	3.13±3.49	5.82±4.01	0.001
APACHE score means± SD	17.49±8.35	14.60±7.07	21.02±8.508	<0.001
Length of hospital stay (days) means± SD	8.37±5.55	9.76±6.14	6.67±4.22	0.005
ICU length of stay (days) means± SD	5.88±3.72	5.71±3.49	6.09±4.02	0.614
***Hemodialysis [n (%)]***				
Yes	18 (18.0%)	10 (18.2%)	8 (17.8%)	1.000
No	82 (82.0%)	45 (81.8%)	37 (82.2%)	
***Mechanical ventilation [n (%)]***				
Invasive	34 (34%)	10 (18.2%)	24 (53.3%)	<0.001
Non invasive	51 (51%)	30 (54.5%)	21(46.7%)	0.547

Age (p=0.031), renal impairment (p=0.014), the presence of two or more complications (p=0.006), AMS (p<0.001), thrombocytopenia (p=0.018), PaO2 (p=0.045), septic shock (p<0.001), duration of MV (p<0.001), high APACHE II score (p<0.001), length of hospitalization (p=0.009), and need for invasive MV (p0.001) were the factors associated with mortality on univariate statistics.

On multivariate statistics, the age (OR 1.054; 95%Cl 1.008-1.103; p=0.021), presence of two or more complications (OR 4.513; 95%Cl 1.178-17.282; p=0.028), septic shock (OR 6.44; 95%Cl 1.547-26.803; p=0.010), duration of MV(OR 1.186; 95%Cl 1.005-1.400; p=0.043), and paO2 (OR 4.514; 95%Cl 1.178-17.282; p=0.004) were all identified to be independent factors related with in-hospital mortality (Table-IV).

**Table-IV:** Univariate and multivariate analysis of the risk factors for in-hospital mortality.

Characteristics	Odds ratios crude	95% Cl	p-value	Odds ratios adjusted	95% Cl	p-value
Age (years)	1.027	1.002-1.052	0.031	1.054	1.008-1.103	0.021
***Number of comorbidities***						
One	1.031	0.235-4.529	0.967			
≥2	1.364	0.351-5.304	0.654	4.513	1.178-17.282	0.028
Cardiovascular diseases	1.633	0.717-3.719	0.242	-	-	-
Renal impairment	2.783	1.226-63.13	0.014	-	-	-
Number of complications ≥2	3.172	1.395-7.210	0.006	-	-	-
Altered mental Status	6.703	2.779-16.167	<0.001	-	-	-
Thrombocytopenia	2.867	1.202-6.836	0.018	-	-	-
Arterial pH	0.820	0.003-2.483	0.151	-	-	-
PaO_2_	0.985	0.970-1.000	0.045	4.513	1.178-17.282	0.004
Shock	0.097	0.037-0.251	<0.001	6.440	1.547-26.803	0.010
MV length (days)	1.235	1.081-1.410	0.002	1.186	1.005-1.400	0.043
APACHEII score	1.114	1.050-1.181	<0.001	-	-	-
Length of hospital stay (days)	0.873	0.788-0.966	0.009	-	-	-
ICU length of stay (days)	1.028	0.924-1.143	0.611	-	-	-
Mechanical ventilation Invasive	5.143	2.088-12.667	<0.001	-	-	-
Mechanical ventilation Non invasive	0.729	0.331-1.607	0.433	-	-	-

Moreover, from multivariate analysis, significant variables were entered in a regression model to see the effect of change. Overall, it was observed that low paO2 alone contributed to the mortality of 4.3% of patients (model 1:R^2^0.043, p=0.038). In the fourth model, the factors which increased the mortality by 38.4% were low paO2, septic shock, the age of the patients, and duration of MV (R^2^0.427, p=0.001).

## DISCUSSION

The main outcome measured in our study was in-hospital mortality. The mean age computed was 60 ±18.01 years, and 64.38 ±15.52 years in non-survivors (p=0.027). Older age is a potential risk factor of mortality on multivariate analysis in our study sample and was also reported as a significant predictor of mortality in similar studies.^15^,^16^ This may be due to an increase in comorbidities, poor performance status and compromised immunity, which are factors commonly found in this age group.^17^

We did not find a significant association of an increased number of comorbidities (more than two) with mortality in our study sample. Consistent findings were observed by Abdel-Aziz et al.^2^ and Lee JH et al.^8^, in different centers. Coronary artery disease (CAD) was the only comorbidity that revealed a significant difference between survivors and non-survivors (p=0.016). This finding is supported widely in the literature, where the presence of diabetes mellitus, CAD and chronic obstructive airway disease all significantly increased hospital mortality.^10^,^16^,^18^-^20^

Complications in patients with CAP considerably increase morbidity, death and healthcare costs. The presence of two or more complications in our patients with SCAP was a significant risk factor for mortality on univariate and multivariate analysis. This finding is supported by the data published by Iqbal N et al.^16^ Septic shock (n=54, 54%) was the most common complication seen, followed by renal impairment (n=53, 53%). Septic shock was observed more in non-survivors (n=37, 82.2%, p<0.001) and was a major risk factor for mortality in our patients, similar to results shown in previous studies.^2^,^8^,^16^,^21^,^22^

Univariate analysis showed age, presence of renal impairment, the presence of two or more complications, AMS, thrombocytopenia,paO2, septic shock, length of MV, a high APACHE II score, duration of hospital stay, and the need for invasive MV were associated with mortality. In addition, the age, presence of two or more complications, septic shock, duration of mechanical ventilation, and paO2 were all identified to be independent factors associated with in-hospital mortality. These findings are consistent with the results published by Abdel-Aziz et al.^2^ and Yoshimoto et al.^21^

Radiologically, we divided both the lungs into four zones. More than half of our patients (54%) had two zones involved. Studies on SCAP have shown an association with bilateral, multilobar involvement and increased in-hospital mortality, 12,23 although this was not observed in our study. Micro-organisms were identified in 50 different specimens and the most common isolate was *A. baumannii*, followed by ESBL-producing *E.coli*. Other studies have identified *Staphylococcus aureus* and *Streptococcus* species as the most common isolated micro-organisms.^1^,^8^,^16^ Viral pneumonia is a recognized cause of mortality in patients with SCAP and three of our patients had positive real-time polymerase chain reaction for influenza A virus subtype H1N1.^24^,^25^ Methicillin-resistant *Staphylococcus aureus* (MRSA) growth was seen in five specimens.

Our main study outcome was in-hospital mortality, which was 45%, which is comparable to other studies carried out worldwide in Egypt, Japan and Korea (with an in-hospital mortality of 49.1%, 48.6%and 56%,respectively).^2^,^8^,^21^This increased number of deaths in our subjects may be due to a majority of patients (85, 85%) requiring resuscitation with IMV and NIV. A high mortality in patients with SCAP could also be because of poor provision of healthcare services at a national level, low socioeconomic status, lack of health awareness and the increased prevalence of health-related misconceptions and beliefs amongst the general public.

### Limitations of the study:

A small sample size, the lack of biochemical infection indicators (e.g., Procalcitonin), and the lack of quantitative culture techniques for pneumonia diagnoses are all noteworthy shortcomings of this study. The primary physician’s discretion was used to conduct the majority of the investigations. Sputum culture was not ordered in all cases. Moreover, data was not available regarding the class, duration and number of antibiotics taken in the community before hospital admission. Since this study was performed in a single hospital, extrapolating the results to other settings should be done with caution.

## CONCLUSION

A high mortality rate was observed in the study population. The frequently isolated etiological agents were *A. baumannii*, followed by ESBL-producing *E.coli*. Age, presence of two or more complications, septic shock, duration of mechanical ventilation and paO2 were all identified to be independent factors associated with in-hospital mortality. Clinical results can be improved by early detection and intervention in these patients who are at a high risk of death. To gain a better understanding of mortality in SCAP patients who develop complications, multicenter studies are needed.

### Authors’ Contributions:

**ARU:** Conception & design.

**AM, SA:** Data collection, literature search and preparation of draft.

**ARU and IA:** Data analysis and interpretation/results.

**ARU, AM, IA:** Manuscript drafting and writing.

**AM, SA and IA:** Language editing/appropriateness, critical revision.

All authors read and approved the final version of the paper. The principal investigator is responsible and accountable for the accuracy or integrity of the work.
